# Interleukin-6 Gene Promoter-572 C Allele may Play a Role in Rate of Disease Progression in Multiple Sclerosis

**DOI:** 10.3390/ijms131013667

**Published:** 2012-10-22

**Authors:** Jun Yan, Jia Liu, Clement Yihao Lin, Australia and New Zealand Multiple Sclerosis Genetics Consortium (ANZGene), Peter A. Csurhes, Michael P. Pender, Pamela A. McCombe, Judith M. Greer

**Affiliations:** 1 The University of Queensland, Centre for Clinical Research, Royal Brisbane & Women’s Hospital, 4029 Brisbane, Australia; E-Mails: jia.liu3@uqconnect.edu.au (J.L.); clement.lin@uqconnect.edu.au (C.Y.L.); p.csurhes@uq.edu.au (P.A.C.); pamela.mccombe@uq.edu.au (P.A.M.); 2 School of Medicine, The University of Queensland, and Department of Neurology, Royal Brisbane & Women’s Hospital, 4029 Brisbane, Australia; E-Mail: m.pender@uq.edu.au

**Keywords:** multiple sclerosis, Interleukin-6, polymorphism, allele, genotype, disease progression

## Abstract

Multiple sclerosis (MS) is an inflammatory demyelinating disease affecting the central nervous system. Although the exact pathogenesis of MS is unknown, it is generally considered to be an autoimmune disease, with numerous genetic and environmental factors determining disease susceptibility and severity. One important mediator of immune responses and inflammation is interleukin-6 (IL-6). Previously, elevated levels of IL-6 in mononuclear cells in blood and in brain tissue from MS patients have been reported. Various polymorphisms in the promoter region of the *IL6* gene have also been linked with IL-6 protein levels. In MS, several small studies have investigated whether two *IL6* promoter polymorphisms (−597 G>A and −174 G>C) correlate with MS susceptibility, but with varying results. In the present study, we analyzed these polymorphisms, together with an additional polymorphism (−572 G>C) in 279 healthy controls and 509 patients with MS. We found no significant differences between MS patients and healthy controls for the different −597 or −174 *IL6* promoter alleles or genotypes. There was a slight reduction in the percentage of individuals with MS who carried a C allele at position −572, although this was not significant after correction for multiple comparisons. Interestingly, however, the −572 C allele showed a significant correlation with the MS severity score, suggesting a possible role in disease progression.

## 1. Introduction

Multiple sclerosis (MS) is a chronic inflammatory demyelinating disease of the central nervous system (CNS) that affects more than 2.5 million people worldwide [1]. The majority of MS patients initially develop MS with a relapsing–remitting disease course (RR-MS) and about 50% of those patients will subsequently develop secondary progressive disease (SP-MS). In 10%–15% of MS cases, however, disease is progressive from the outset and is known as primary progressive MS (PP-MS) [2]. RR-MS affects females more frequently than males (3:1), but in PP-MS, males and females are equally affected [3]. One of the hallmarks of MS is the unpredictability of the rate of progression of disease; some patients rapidly progress, whereas others may go many years without accumulation of significant disability.

The causes of MS are uncertain, and might not be identical in all individuals. It is believed that, in the majority of patients, the disease is maintained and driven by the development of autoimmune reactivity directed against myelin or neuronal components [4,5]; however, the exact precipitating events that allow the development of autoimmunity are not clear, and it is likely that both an underlying genetic susceptibility and exposure to one or more environmental triggers are required. Recent studies have identified numerous gene polymorphisms that appear to relate to MS susceptibility [5–7], although for many of these genes the effects are relatively small. Little is known, however, about genes that affect MS severity or the clinical course of MS.

Several studies have investigated whether two promoter region polymorphisms in the gene encoding interleukin 6 (IL-6), a pleiotropic cytokine that is an important mediator of many inflammatory processes, including induction of the acute phase response and differentiation of lymphocytes and monocytes, might enhance susceptibility to MS [8–11]. Increased levels of IL-6 have been detected in mononuclear cells in the blood and cerebrospinal fluid (CSF) [12–14] and in brain tissue of patients with MS [15]. Furthermore, studies from both human MS patients [13] and from mouse models of MS [16] suggest that IL-6 levels may correlate with disease severity. Because it is known that some of the *IL6* promoter region polymorphisms can affect IL-6 levels, it is of interest to investigate the relationship between these polymorphisms, and MS susceptibility and severity.

The two *IL6* polymorphisms that have thus far been investigated in MS are −597 G/A (rs 1800797) and −174 G/C (rs1800795); these are in very strong linkage disequilibrium [17]. The −174 C allele can suppress IL-6 transcription [18], but less is known of the functional correlates of other *IL6* promoter polymorphisms. In the MS studies, two groups reported no linkage between the −597 and −174 *IL6* promoter polymorphisms and MS [8,9], whereas the other 2 studies did report significant correlations, but with different alleles/genotypes [10,11]. In all of these studies, the numbers of patients were fairly low, and few PP-MS patients were tested. We have not been able to ascertain whether rs1800795 and rs1800797 were on the chips used in a large recent international MS GWAS [5], but have confirmed that they were not represented on the chips used in the ANZGene MS GWAS [6].

In kidney allograft survival studies, it has been found that the −597 and −174 *IL6* promoter polymorphisms can operate cooperatively with another *IL6* promoter polymorphism at −572 to influence graft survival [19], suggesting that it might be necessary to investigate the broader *IL6* haplotype in order to determine if *IL6* promoter polymorphisms are of functional relevance. The −572 G>C polymorphism (rs 1800796) has not previously been studied in MS. The aim of the present study was therefore to investigate the *IL6* promoter polymorphisms −597, −572 and −174 in a large group of MS patients and controls, and establish their relationship to MS susceptibility and progression.

## 2. Results and Discussion

### 2.1. Allelic and Genotypic Frequency of the *IL6* Promoter Region Polymorphisms

Genomic DNA samples from patients with MS (*n* = 509) and healthy controls (*n* = 279) (see Table 1 for details of participants) were sequenced to determine the alleles and genotypes present in each individual (Table 2).

**Figure 1:**
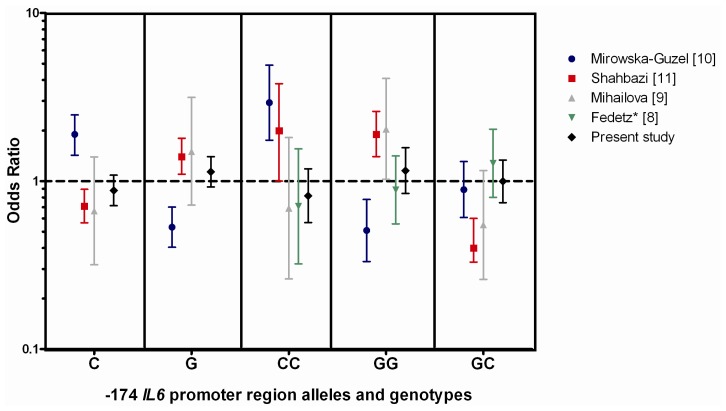
Meta analysis of the odds ratios in the five studies that have investigated −174 *IL6* promoter region polymorphisms in MS. * This study inferred the −174 genotypes from the −597 genotype.

There were no differences in the frequency of any of the *IL6* promoter region polymorphisms between MS patients and controls, apart from a slight decrease in the frequency of the −572 C allele and CC genotype in patients with MS compared to healthy controls; these were not significant after correction for multiple comparisons. Interestingly, the decrease was not seen in patients with PP-MS. Our results are in striking contrast to those of both Shahbazi *et al.*, who found an increased risk for MS associated with the G allele of −174 in an Iranian cohort [11], and of Mirowska-Guzel *et al*, who found increased risk of MS to be associated with the C allele of −174, particularly with the CC genotype in a Polish cohort [10]. Whether these differences are due to differences in the ethnicity of the populations, or to some other factor, remains to be determined. A meta analysis of the −174 *IL6* promoter region region genotype findings from the 4 previous studies in MS and the present study is shown in Figure 1. The numbers of samples in each of these studies were: Mirowska-Guzel *et al*. [10] 228 MS and 193 controls; Shahbazi *et al*. [11] 345 MS and 426 controls; Mihailova *et al*. [9] 55 MS and 86 controls; Fedetz *et al*. [8] 131 MS and 157 controls; present study 509 MS and 279 controls.

### 2.2. −572 *IL6* Promoter Region Genotypes, but not −172 Genotypes, Associate with MS Severity

**Figure 2:**
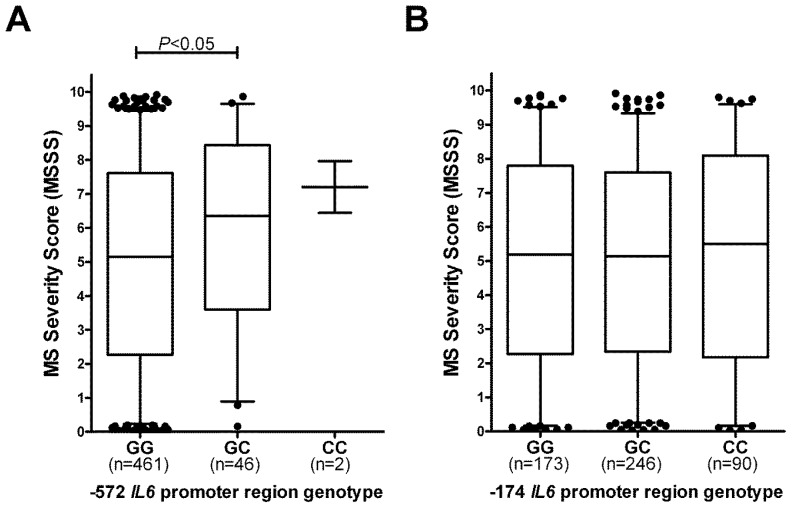
(**A**) Distribution of MS Severity Scores (MSSS) according to patient −572 genotype. Each bar on the graphs represents the median and interquartile range. The whiskers represent the 5th and 95th percentiles, and outliers are shown as black circles above and below the plots; (**B**) Distribution of MSSS in patients subdivided on basis of −174 genotype.

We next analyzed whether carriage of the different *IL6* promoter region genotypes correlated with the severity of MS. The MS Severity Score (MSSS) [20] takes into account both the level of disease disability and the duration of disease, and is scored from 0 to 10, with 10 being death from MS. Patients with the −572 GC genotype overall showed a significantly higher median MSSS than did those with the GG genotype (*p* < 0.05) (Figure 2A). Only 2 of the 509 MS patients carried the CC genotype, and so no conclusions can be drawn regarding the effect of this on MS, although those two individuals did have a relatively high MSSS. In contrast, there was no difference in the median or IQR of the MSSS of patients with the different −174 genotypes (Figure 2B).

#### 2.2.1. Interaction of Gender with Genotype

**Figure 3:**
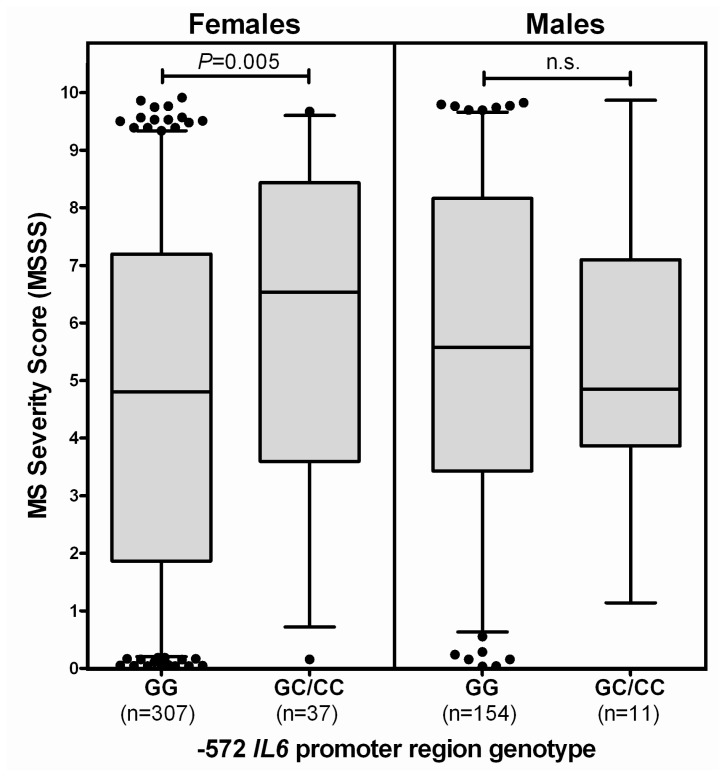
MS Severity Scores in females and males, by −572 *IL6* genotype.

Because there were significant differences in the proportion of females with RR-MS compared to the other types of MS in our study, and because previous studies have identified the −174 G>C polymorphism as a 17β-estradiol sensitive site in females with type 1 diabetes [21], we next analyzed whether carriage of the different *IL6* promoter region genotypes differed according to the gender of the patients. For the −572 promoter polymorphism, as there was only 1 female and 1 male who had the CC genotype, GC and CC genotypes were considered together. Females with a GC/CC genotype showed a significantly (*p* = 0.005) elevated MSSS compared to females of GG genotype (Figure 3). For the other two *IL6* promoter polymorphisms (−597 G>A and −174 G>C), there were no differences between males and females for the median MSSS scores (not shown).

**Figure 4:**
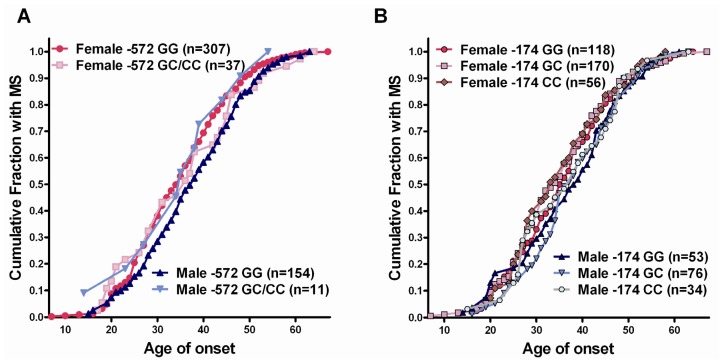
Cumulative fraction of patients carrying different *IL6* promoter genotypes *vs.* age of onset of MS. (**A**) Patients grouped according to −572 *IL6* genotype; (**B**) Patients grouped according to −174 *IL6* genotype.

Previously, it has been shown in type 1 diabetes that the −174 *IL6* genotype is associated with the age of onset of disease in females, but not males [21]. Therefore, we looked at whether the genotype was associated with the age of onset of disease for the −572 and −174 genotypes. For the −572 genotypes, as there was only 1 female and 1 male who had the CC genotype, GC and CC genotypes were considered together. It has been established previously that females typically have a slightly earlier age of onset of MS than do males [22], and this was reflected in the analysis of the genotypes, where females typically developed MS 2–3 years earlier than males, particularly for age of onset between 25 and 50 years (Figure 4). This is most clearly seen when comparing the GG genotypes for −572, where the line for the females (particularly for age of onset between 25 and 50) is to the left and higher than the male GG group. There was no association between genotype and age of onset of MS in females. There appeared to be a slight decrease in the time to disease onset in males who carried the −572 GC or CC genotypes (Figure 4A); however, there were only 11 males in this group, which precludes drawing any firm conclusions. For the −174 *IL6* genotypes, there was no significant effect of the different genotypes on age of onset of MS (Figure 4B).

#### 2.2.2. The −572 *IL6* GC Genotype Association with MS Severity is Most Noticeable in Patients Who Initially Have RR-MS

**Figure 5:**
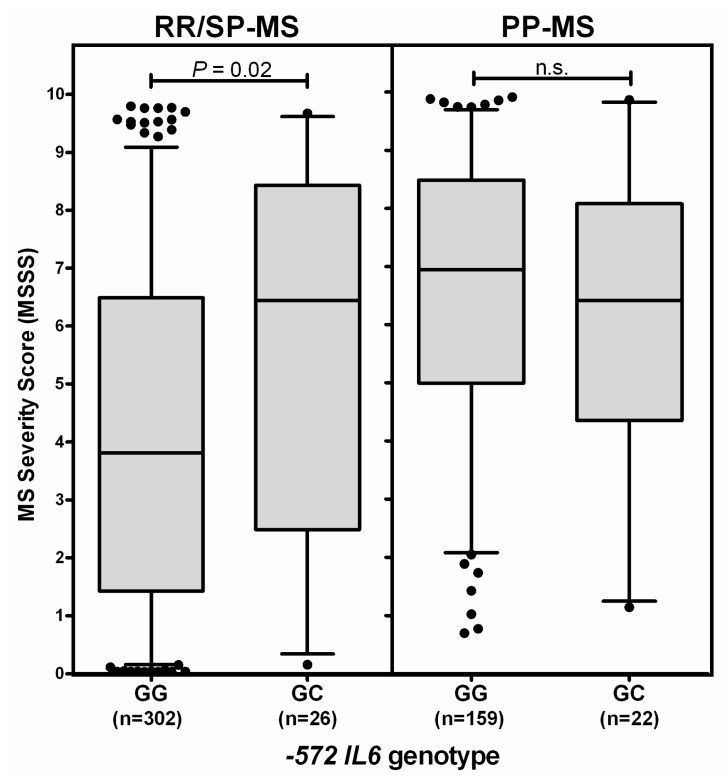
MS Severity Scores in patients who initially have a RR-MS course, compared to a PP-MS course, by −572 *IL6* genotype.

The ratio of females to males with MS is significantly different in the group of patients who initially have RR-MS (*i.e.*, the RR-MS + SP-MS groups), compared to patients who have PP-MS. Because we found that disease severity was elevated in females, but not males, of the −572 GC/CC genotype (Figure 2), and because the allele frequency of the −572 C allele was somewhat reduced in MS compared to controls, particularly in the patients with RR-MS (Table 1), we therefore next investigated whether the MSSS differed in patients with different −572 genotypes who were subdivided on the basis of disease course. There was a significant difference between the MSSS of GG and GC/CC patients who initially had a RR course of disease, but not for those who had a primary progressive disease course (*p* = 0.02; Figure 5). We considered whether the differences between GG and GC carriers in the RR/SP group could be due to a higher proportion of patients with SP-MS in the GC group; however, this does not appear to be the case, as there was no significant difference in the proportion of SPMS patients in the GC group compared to the proportion of SP-MS patients in the GG group (57.7% *vs.* 48.7%; *p* = 0.53).

#### 2.2.3. Interaction of HLA Type with *IL6* Promoter Region Genotype

One of the recent publications that found significant differences in the −174 *IL6* promoter region genotypes between patients and controls were intensified in the subgroup of patients who carry HLA-DRB1*1501 [11]. In Caucasian populations, DRB1*1501 occurs approximately twice as frequently in people with MS than in healthy individuals. We therefore analyzed whether carriage of DRB1*1501 by MS patients was associated with the frequency of the different genotypes or with the MSSS in patients with different genotypes. Four hundred and ninety-one MS patients (96.5% of total) were typed to determine whether or not they carried HLA-DRB1*1501. Fifty-seven percent of all MS patients were positive for DRB1*1501. The percentage of DRB1*1501 positive MS patients was only marginally different between RR/SP-MS (57.6%) and PP-MS (55.9%). The ratio of DRB1*1501 positive to DRB1*1501 negative MS patients did not change significantly, irrespective of the *IL6* alleles or genotypes of the patients (data not shown). Similarly, when the MSSS was compared between DRB1*1501 positive and negative individuals, stratified according to the −572 or −174 *IL6* promoter region genotypes, there was no effect attributable to the HLA type (not shown). Therefore, in this sample, we were unable to confirm the previous results of an effect of DRB1*1501 on the *IL6* genotype.

#### 2.3. Haplotypes of the *IL6* Promoter Region Polymorphisms

**Figure 6:**
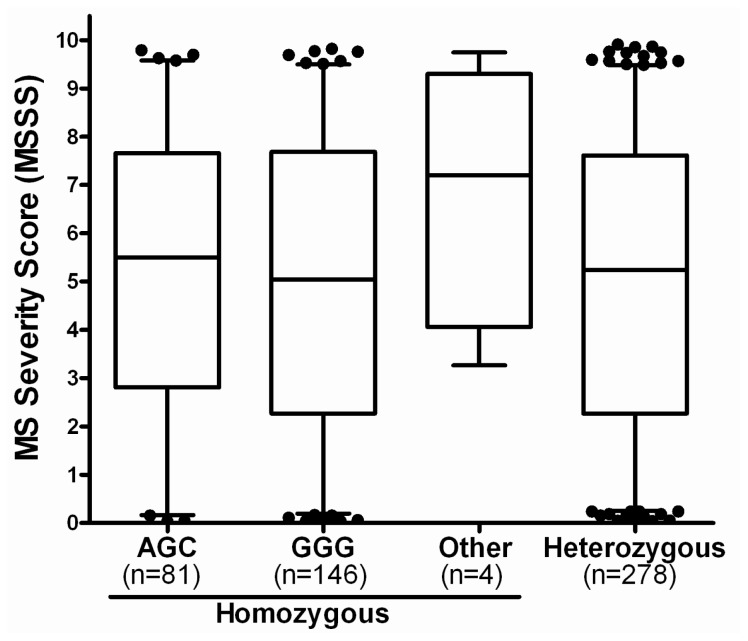
MSSS of patients with different *IL6* promoter region haplotypes. There were no significant differences between patients with different haplotypes.

One of the limitations of the method employed for DNA sequencing in this paper is that it is not possible to accurately assign haplotypes for heterozygous genotypes. However, a substantial proportion of the MS (45.4%) and healthy control (44.4%) populations are homozygous across each genotype, and therefore their haplotypes could be correctly assigned. We have therefore analyzed the frequency of carriage of these homozygous haplotypes in healthy controls and patients with MS (Table 3). There were no significant differences between healthy controls and MS patients for these haplotypes. Futhermore, there were no significant differences in the MSSS of patients of these different haplotypes, or between patients who were homozygous or heterozygous (Figure 6).

## 3. Experimental Section

### 3.1. Patients and Controls

Our study was reviewed and approved by the Royal Brisbane and Women’s Hospital Health Service District Office of the Human Research Ethics Committee and by the Medical Research Ethics Committee, The University of Queensland, Brisbane, Australia. Blood or saliva was collected, after informed consent was obtained, from 279 healthy controls and 509 patients with MS. The age, sex and, for the MS patients, clinical course of disease and MSSS are shown in Table 1. The MSSS is an algorithm which relates scores on the Kurtzke Expanded Disability Status Scale (EDSS) to the distribution of disability in patients with comparable disease durations [20]. The MSSS is scored from 0.01 (least severe) to 9.99 (most severe). The MS patients were all recruited by the Australia and New Zealand Multiple Sclerosis Genetics Consortium (ANZgene) and met the 2005 revised McDonald criteria for MS [23]. Healthy controls were recruited from Royal Brisbane and Women’s Hospital or University of Queensland staff.

### 3.2. Genomic DNA Isolation and Sequencing

Genomic DNA was extracted from healthy control blood samples using a Qiagen blood DNA extraction kit (Qiagen, Australia). Genomic DNA samples from either blood or saliva of MS patients were provided by ANZgene consortium and were prepared as previously described [6]. The DNA concentration was determined using a NanoDrop™ 1000 Spectrophotometer (Thermo Scientific, Waltham, MA, USA). DNA sequencing was done at the Australian Genomic Research Facility, Brisbane, Queensland, Australia or at the Beijing Genomic Institute, ShenZhen, China. PCR primers used for sequencing IL-6 −597 G>A and −572 G>C polymorphisms were 5′-TGAGGCTAGCGCTAAGAAGC-3′ (forward) and 5′-CTGGGAGGATTCCCAAGG-3′ (reverse). For sequencing the −174 G>C polymorphism the primers were 5′-AGACATGCCAAAGTGCTGAGT-3′ (forward) and 5′-GCTCCTGGAGGGGAGATAGA-3′ (reverse).

### 3.3. Data Analysis

Comparisons of allele and genotype frequencies between healthy controls and MS patients (and subgroups of MS) was made by χ^2^ analysis. For comparison of MSSS values in patients of different genotypes, all data were first checked to determine if they were normally distributed. The MSSS values were not normally distributed, and therefore were analyzed using the non-parametric Mann-Whitney test for comparison of 2 populations or the Kruskal-Wallis analysis of variance method for comparison of 3 or more populations.

## 4. Conclusions

The −572 *IL6* promoter region C allele appears to correlate with the MSSS, which combines measures of the disease severity and disease duration, even though *IL6* promoter region polymorphisms do not appear to associate strongly with susceptibility to MS. A caveat to this is that because the sample size is still relatively small, and the frequency of the C allele is relatively low, this observation will need to be replicated to confirm whether *IL6* promoter region polymorphisms are indeed a predictor of MS severity.

The correlation between the −572 *IL6* promoter region C allele and MS severity score is particularly noticeable in female patients, when patients are divided according to gender, which is of interest, as estrogen is a well-known modulator of *IL6* gene expression.

The clinical subtype of MS also affected the correlation between MSSS and −572 *IL6* genotype. This could be because the percentage of females in the RR-MS group was significantly greater than the percentage of females in the PP-MS group, or for other reasons not yet elucidated.

It is not known whether the −572 *IL6* promoter region polymorphism influences IL-6 levels; further *in vitro* functional assays will be required to prove whether the −572 C allele alone, or haplotypes containing this allele, regulate the expression of IL-6.

We were unable to reproduce previous studies suggesting that polymorphisms at −174 of the *IL6* promoter region correlate with susceptibility to MS [10,11]. The reasons for this could be due to differences in the ethnicity of patients in those studies and the current study, the sample size used in the studies, or to other factors as yet undetermined.

## Figures and Tables

**Table 1:** Details of participants.

Group	*n*	% Female	Age (Mean ± SE)	MSSS * median (IQR)
Healthy controls	279	63.1%	42 ± 1.0	not applicable
Multiple sclerosis				
All	509	68.4%	52.8 ± 0.5	5.24 (2.33–7.66)
RR-MS	156	82.1% †	46.6 ± 0.9	1.73 (0.49–3.42)
SP-MS	172	69.2%	53.4 ± 0.8	6.28 (4.13–8.20)
PP-MS	181	56.9%	57.6 ± 0.7	6.90 (4.82–8.49)

* The MS Severity Scores (MSSS) [20] were not normally distributed, and are therefore presented as the median and interquartile range (IQR).

† The proportion of females in the RR-MS group was significantly different from that in the healthy control group (*p* = 3.5 × 10^−5^), the SP-MS group (*p* = 0.007) and the PP-MS group (*p* = 7 × 10^−7^).

**Table 2:** Results of allelic and genotypic tests for the *IL6* promoter polymorphisms.

Allele/genotype		Healthy controls	Multiple sclerosis					*P*_uncorrected_ *vs.* healthy controls

			All	RR-MS	SP-MS	PP-MS		
−597	G	318 ^(57.0%)^	607 ^(59.6%)^	182 ^(58.3%)^	209 ^(60.8%)^	216 ^(59.7%)^	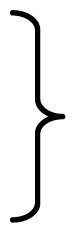	n.s.
	A	240 ^(43.0%)^	411 ^(40.4%)^	130 ^(41.7%)^	135 ^(39.2%)^	146 ^(40.3%)^		
	GG	94 ^(33.7%)^	181 ^(35.6%)^	54 ^(34.6%)^	64 ^(37.2%)^	63 ^(34.8%)^		n.s.
	GA	130 ^(46.6%)^	245 ^(48.1%)^	74 ^(47.4%)^	81 ^(47.1%)^	90 ^(49.7%)^		n.s.
	AA	55 ^(19.7%)^	83 ^(16.3%)^	28 ^(17.9%)^	27 ^(15.7%)^	28 ^(15.5%)^		n.s.

−572	G	517 ^(92.7%)^	968 ^(95.1%)^	301 ^(96.5%)^	328 ^(95.3%)^	339 ^(93.6%)^	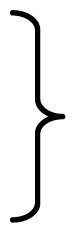	0.047 (all MS);
	C	41 ^(7.3%)^	50 ^(4.9%)^	11 ^(3.5%)^	16 ^(4.7%)^	23 ^(6.4%)^		0.03 (RR-MS)
	GG	244 ^(87.5%)^	461 ^(90.6%)^	145 ^(92.9%)^	157 ^(91.3%)^	159 ^(87.8%)^		n.s.
	GC	29 ^(10.4%)^	46 ^(9.0%)^	11 ^(7.1%)^	14 ^(8.1%)^	21 ^(11.6%)^		n.s.
	CC	6 ^(2.2%)^	2 ^(0.4%)^	0 ^(0%)^	1 ^(0.6%)^	1 ^(0.6%)^		0.047 (all MS)

−174	G	307 ^(55.0%)^	592 ^(58.2%)^	176 ^(56.4%)^	204 ^(59.3%)^	212 ^(58.6%)^	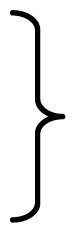	n.s.
	C	251 ^(45.0%)^	426 ^(41.8%)^	136 ^(43.6%)^	140 ^(40.7%)^	150 ^(41.4%)^		
	GG	86 ^(30.8%)^	173 ^(34.0%)^	50 ^(32.1%)^	62 ^(36.0%)^	61 ^(33.7%)^		n.s.
	GC	135 ^(48.4%)^	246 ^(48.3%)^	76 ^(48.7%)^	80 ^(46.5%)^	90 ^(49.7%)^		n.s.
	CC	58 ^(20.8%)^	90 ^(17.7%)^	30 ^(19.2%)^	30 ^(17.4%)^	30 ^(16.6%)^		n.s.

n.s.: not significant.

**Table 3:** Frequency of *IL6* promoter region haplotypes, for those individuals who had identical haplotypes on each DNA strand.

Homozygous haplotype (−597, −572, −174)	Healthy controls (*n* = 124)	Multiple sclerosis				*P*_uncorrected_ *vs.* healthy controls

		All (*n* = 231)	RR-MS (*n* = 72)	SP-MS (*n* = 84)	PP-MS (*n* = 75)	
GGG	85 ^(68.5%)^	146 ^(63.2%)^	45 ^(62.5%)^	53 ^(63.1%)^	48 ^(64.0%)^	n.s.
ACG	39 ^(31.5%)^	81 ^(35.1%)^	27 ^(37.5%)^	29 ^(34.5%)^	25 ^(33.3%)^	n.s.
Other	0 ^(0%)^	4 ^(1.7%)^	0 ^(0%)^	2 ^(2.4%)^	2 ^(2.7%)^	n.s.
